# Confirmed complete response to nivolumab for advanced gastric cancer with peritoneal dissemination: a case report

**DOI:** 10.1186/s13256-021-03200-x

**Published:** 2021-12-21

**Authors:** Tomoya Takami, Koji Yasuda, Nozomi Uozumi, Yutaka Musiake, Hiroshi Shintani, Naoki Kataoka, Tomoyuki Yamaguchi, Shinichiro Makimoto

**Affiliations:** Department of General Surgery, Kishiwada Tokushukai Hospital, 4-27-1 Kamoricho, Kishiwada-shi, Osaka-fu 596-0042 Japan

**Keywords:** Nivolumab, Advanced gastric cancer, Complete response, Prognosis

## Abstract

**Background:**

Recent advances in cancer immunotherapy have been remarkable, with many reports on the clinical effects of immune checkpoint inhibitors. Nivolumab has been covered by the national health insurance in Japan as a third-line agent for advanced and recurrent gastric cancer since September 2017. The objective response rate for nivolumab for gastric cancer is 11.2%. However, patients’ quality of life during this treatment has not been examined. Here, we report a case in which multidisciplinary treatment, including with nivolumab, resulted in long-term survival and improved quality of life.

**Case presentation:**

A 70-year-old Asian woman was referred for surgery for gastric cancer. Postoperative pathological examination revealed peritoneal dissemination, and the patient was diagnosed with stage IV gastric cancer. Therefore, she was treated with S-1 and cisplatin based on negative immunohistochemical staining of resected specimens for human epidermal growth factor receptor 2. However, owing to instability and adverse events, treatment was subsequently changed to S-1 monotherapy. Two years after changing to S-1 monotherapy, she developed recurrence of peritoneal dissemination and was treated with docetaxel. Radiation therapy was also used because the recurrent lesions were local. However, 6 months later, new peritoneal dissemination and lymph node metastasis were observed and nivolumab was started. Subsequent abdominal computed tomography revealed a marked reduction in the disseminated nodules and lymphadenopathy. After 54 cycles of nivolumab, the lesions had disappeared completely. The patient has not developed side effects, including immune-responsive adverse events, has improved quality of life, and is returning to work. She is currently taking nivolumab, and there is no evidence of recurrence approximately 3 years after starting nivolumab.

**Conclusions:**

Nivolumab may have beneficial effects in some patients with advanced or recurrent gastric cancer. Although the prognosis for gastric cancer and peritoneal dissemination is poor, multidisciplinary treatment that includes nivolumab may lead to long-term survival.

## Background

Cancer immunotherapy has recently undergone remarkable developments and is effective against various types of cancer. The mechanism of action of immunotherapy is different from that of conventional antineoplastic agents. Immune checkpoint inhibitors, such as anti-cytotoxic T-lymphocyte-associated protein 4 and anti-programmed cell death 1 (PD-1) antibodies, are currently being used clinically for lung cancer therapy [1, 2]. However, while developments in drug therapy can be expected to prolong the survival of unresectable advanced/recurrent gastric cancer, complete response (CR) is rarely achieved. We report a patient who achieved CR with nivolumab treatment.

## Case presentation

Our patient was a 70-year-old Asian woman who visited the hospital with a complaint of epigastric pain. She had no notable family history and no history of smoking or drinking. She underwent upper endoscopy, and was diagnosed with gastric cancer; therefore, she was referred for surgery.

**Fig. 1:**
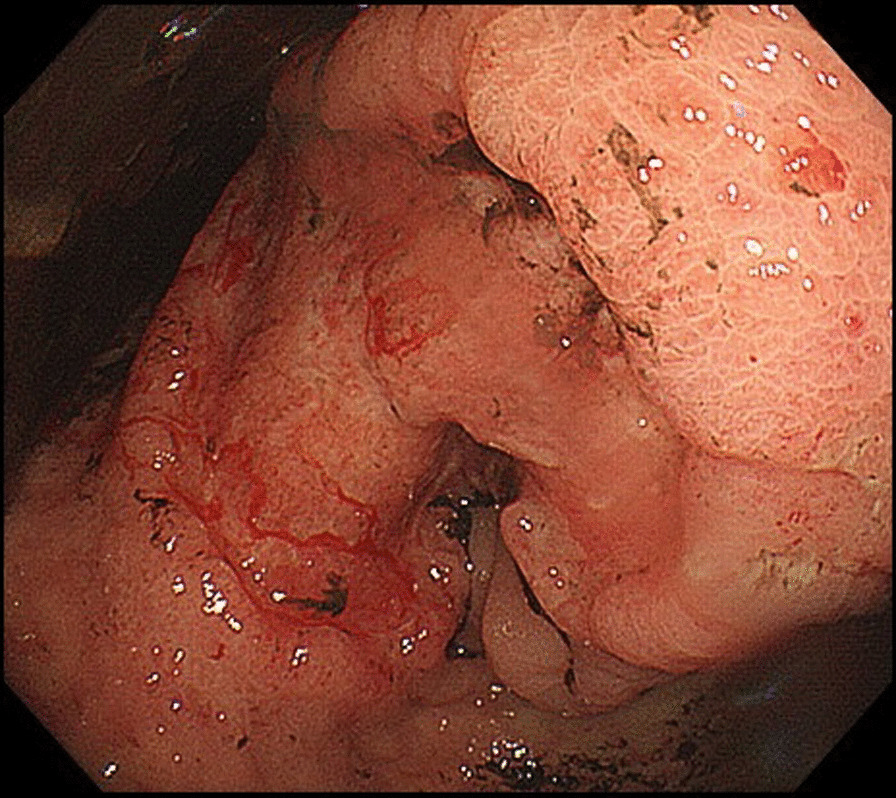
Gastrointestinal fiberoptic endoscopy. A type 3 tumor was found on the lesser curvature side of the angular incisure

**Fig. 2:**
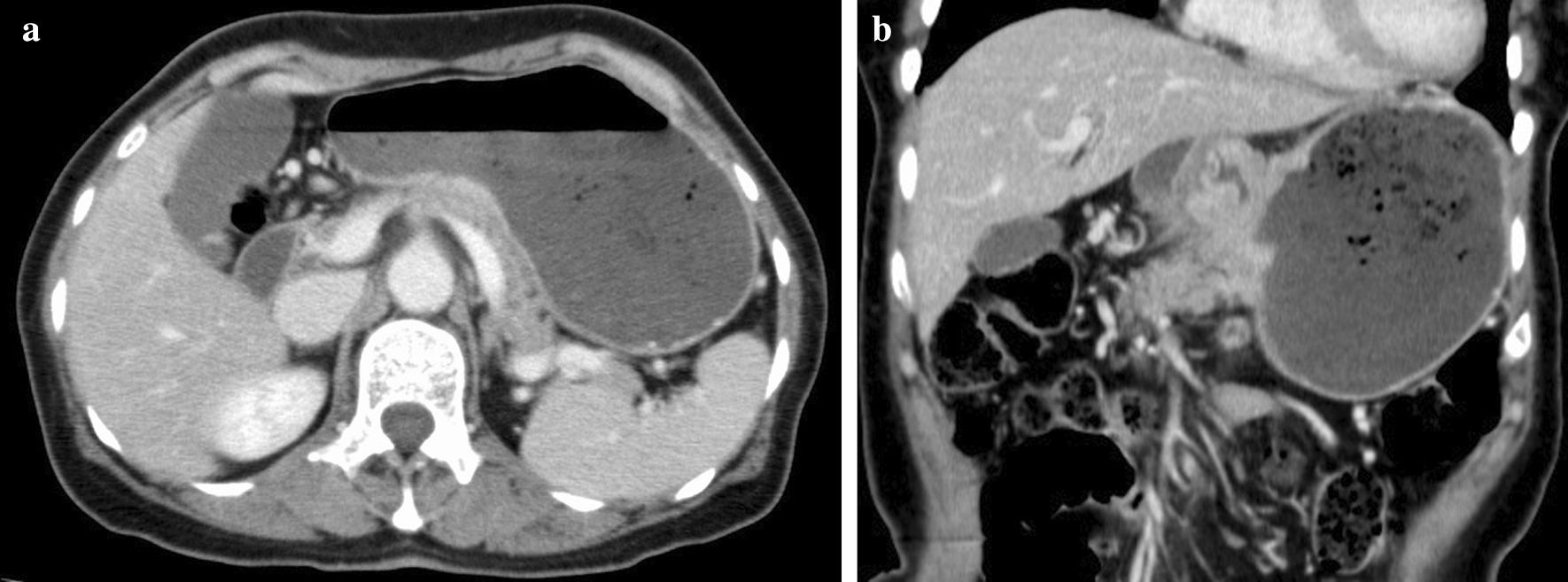
**a** Computed tomography (coronal plane). Suspected infiltration of the tumor into the pancreas. **b** Computed tomography (axial view of the same location as in Fig. 1-1)

The patient was 153 cm tall, weighed 44 kg, and had a body mass index (BMI) of 18.8 kg/m^2^. Her abdomen was flat and soft, and Virchow’s lymph nodes were not palpable. The patient’s hemoglobin concentration was 6.1 g/dL, blood urea nitrogen concentration was 25.8 mg/dL, carcinoembryonic antigen (CEA) was 0.9 ng/mL, and carbohydrate antigen (CA)19-9 was 9.6 U/mL. Upper endoscopy showed a type 3 tumor on the lesser curvature side of the gastric angle (Fig. 1), and biopsy revealed a group 5, well-differentiated adenocarcinoma. Computed tomography (CT) of the chest and abdomen showed wall thickening with contrast enhancement on the lesser curvature side of the angular incisure, part of which was in contact with the pancreas. Gastric cancer may have invaded the pancreas because the fat at the border with the pancreas had disappeared. In addition, the lymph nodes on the lesser curvature and the liver hilum were slightly swollen (Fig 2a and b). A positron emission tomography (PET) scan showed accumulation only in tumors (SUV max 8.0), but not in lymph nodes or other organs. Based on these findings, the preoperative pathological diagnosis was T4b N1 M0 stage IIIb, and surgery was indicated.

Intraoperative findings showed no ascites or peritoneal dissemination. However, because gastric cancer had invaded the pancreas, total gastrectomy, splenectomy, and resection of the pancreatic tail were performed. The postoperative course was uneventful, and the patient was discharged 18 days after surgery.

**Fig. 3:**
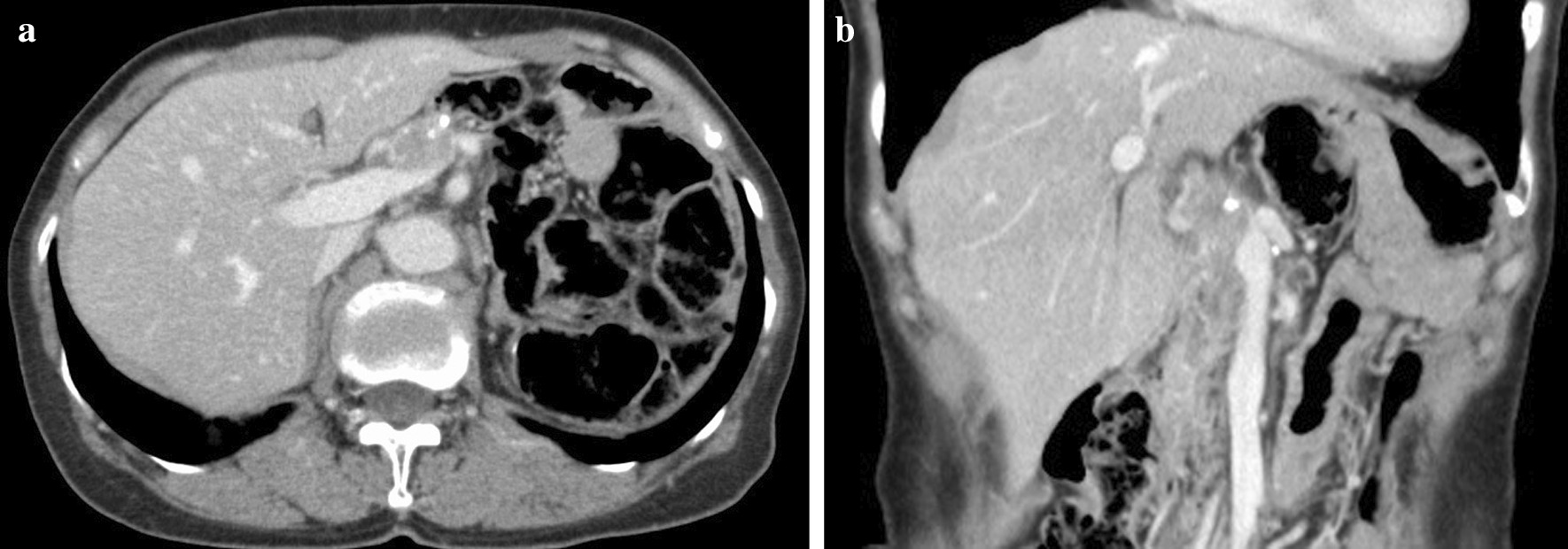
**a** Computed tomography (coronal plane). A nodule measuring approximately 20 mm is visible on the pancreatic stump. **b** Computed tomography (axial view of the same location as in Fig. 3-1)

**Fig. 4:**
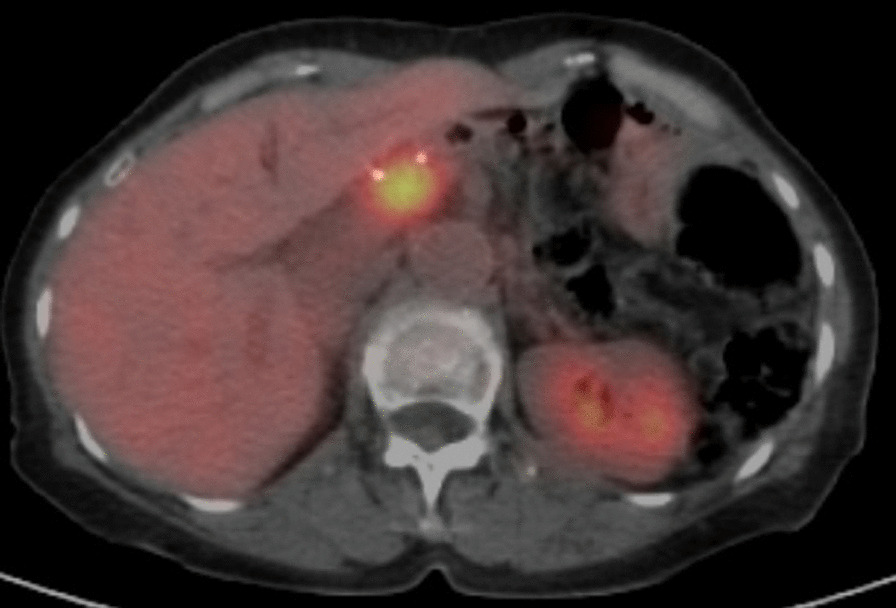
Positron emission tomography-computed tomography. Accumulation is observed in the nodule image

**Fig. 5:**
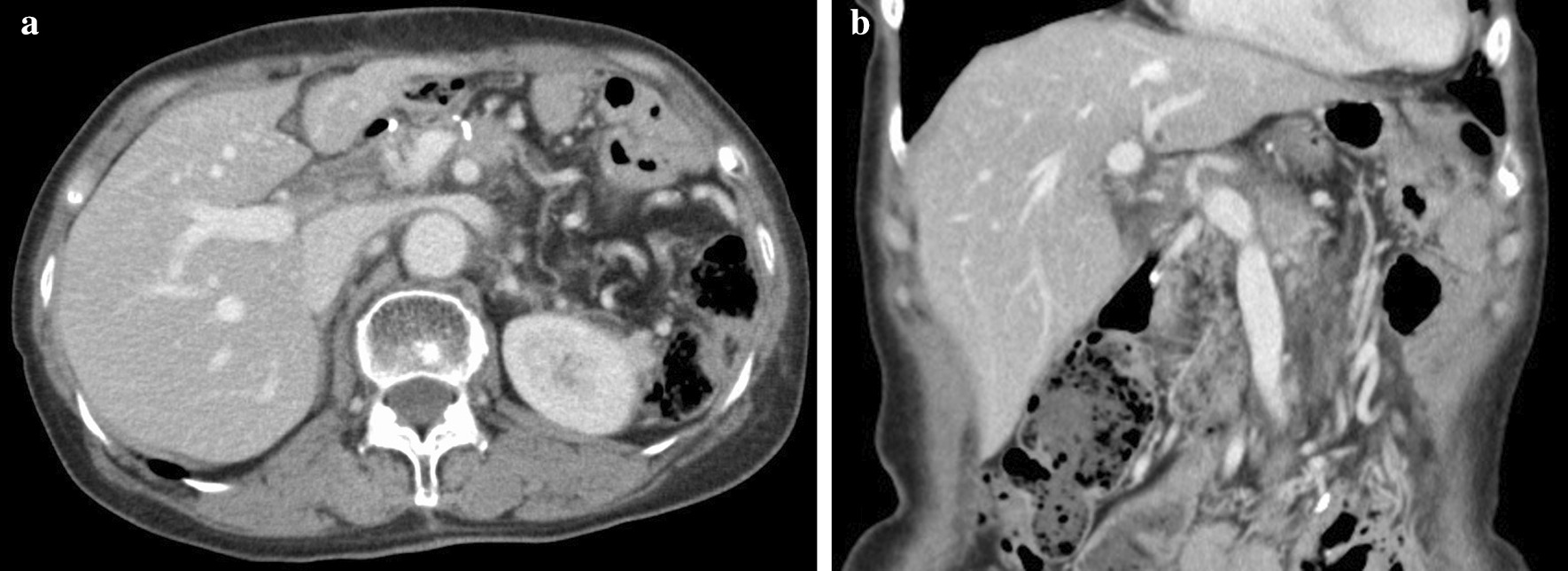
**a** Computed tomography (coronal plane). A nodule image measuring approximately 13 mm is visible. **b** Computed tomography (axial view of the same location as in Fig. 5-1)

**Fig. 6:**
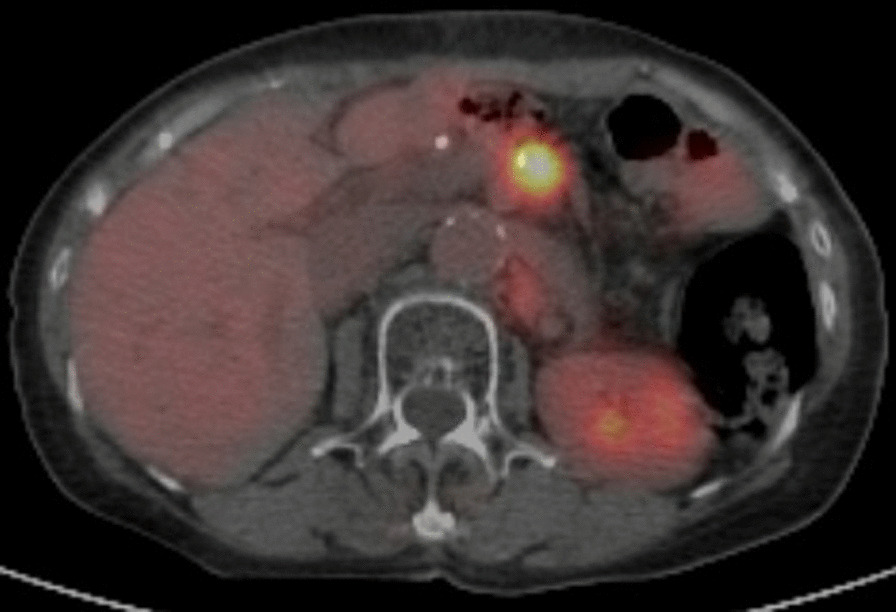
Positron emission tomography-computed tomography. Accumulation is observed in the nodule image

**Fig. 7:**
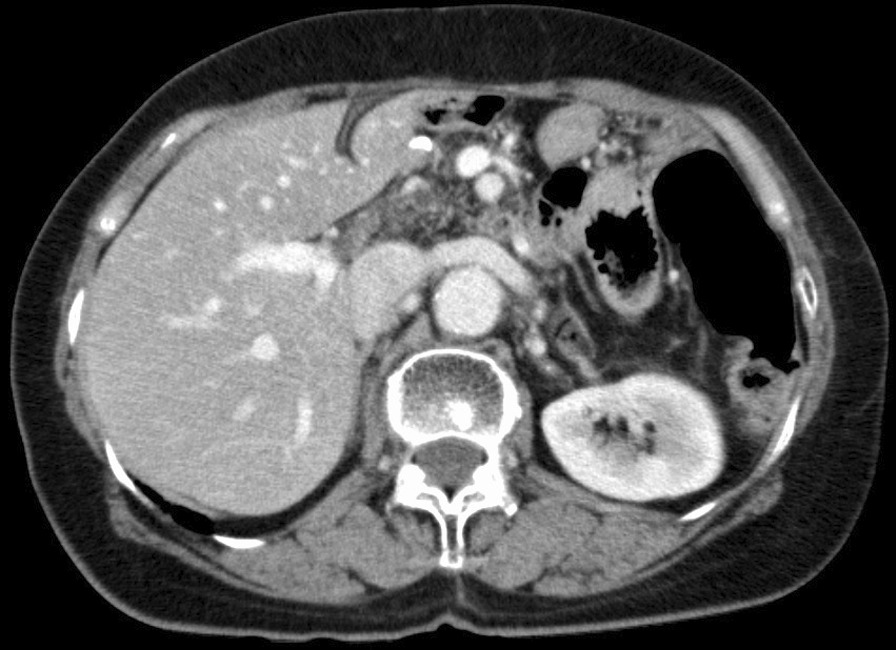
Computed tomography (coronal plane). No nodules can be identified

**Fig. 8:**
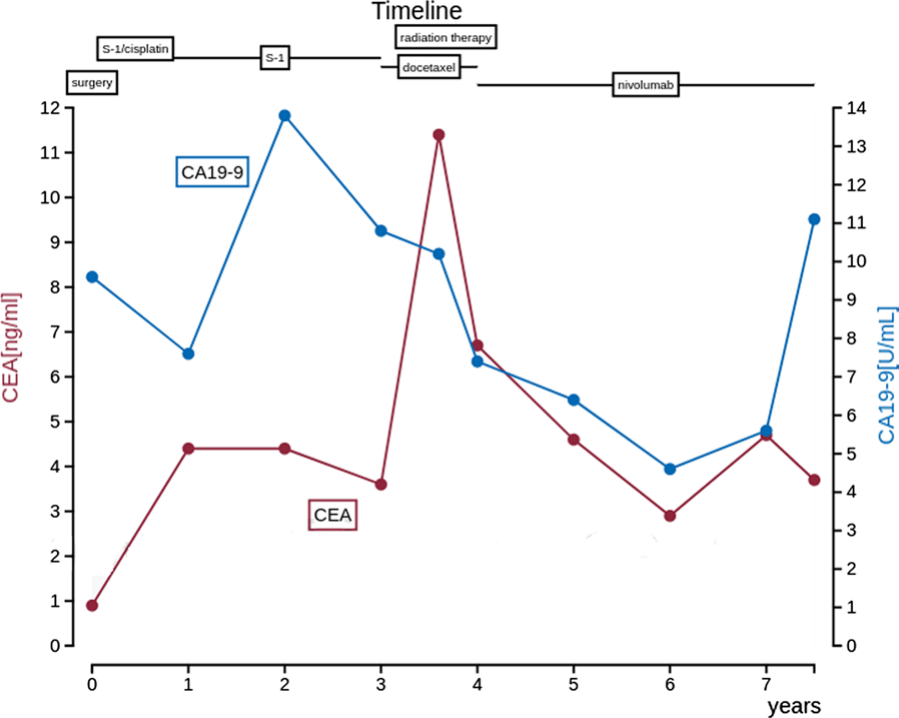
Postoperative course

Postoperative pathological examination revealed small disseminated nodules in the resected specimen. The final diagnosis was T4bN3aM1, stage IV, according to the 8th Union for International Cancer Control (UICC) TNM classification [3]. Immunohistochemically, the tumor was negative for human epidermal growth factor receptor 2 (HER2). Therefore, we started combination therapy with S-1 and cisplatin as first-line chemotherapy. S-1 was administered orally at a dose of 40 mg twice daily for the first 3 weeks in a 5-week cycle, with an intravenous dose of 60 mg/m^2^ cisplatin on the seventh day of each cycle. The therapeutic effect was judged according to the RECIST guidelines, version 1.1; adverse events were recorded in accordance with CTCAE criteria, version 4.0 [4, 5]; and no recurrence was observed during the first 6 months of treatment. However, owing to a grade 3 loss of appetite, the patient experienced marked weight loss to 37 kg; therefore, cisplatin administration was discontinued after seven courses, and therapy was changed to S-1 monotherapy. After the change, her condition stabilized, but a CT scan 3 years after surgery showed a 20 mm nodule on the stump of the resected pancreas (Fig. 3a and b). The CEA concentration was 3.6 ng/mL and the CA19-9 concentration was 10.8 U/mL, which were within the normal ranges; however, positron emission tomography-CT (PET-CT) showed contrast accumulation at the pancreatic stump (Fig. 4). Therefore, we diagnosed recurrence owing to dissemination. Because it was a treatment for local recurrence, radiation therapy was administered for the nodule, and chemotherapy with docetaxel was started at the patient’s request. She received docetaxel 60 mg/m^2^ intravenously on the first day of the 21-day cycle. Although side effects such as malaise were observed, the nodule tended to shrink with continued administration. However, after 14 courses of systemic treatment (4 years after surgery), abdominal CT showed new peritoneal dissemination and lymphadenopathy. The largest disseminated nodule was a 13 mm contrast-enhanced nodule around the superior mesenteric artery that increased in size over time (Fig. 5a and b). The CEA concentration increased to 11.4 ng/mL and the CA19-9 concentration increased to 10.8 U/mL. PET-CT showed accumulation in the pancreatic stump and in the periaortic lymph nodes. Accumulation was also seen in part of the abdominal wall (Fig. 6). With dissemination, the patient’s symptoms progressed, and she was diagnosed with lymph node recurrence and peritoneal dissemination; therapy was changed to intravenous nivolumab 3 mg/kg every 2 weeks. Owing to a subsequent change in the dosage standard, the dose was increased to 240 mg. After starting immunotherapy, CT scans were done every 3–4 months. Nodules or lymph node swelling were not identified on CT 54 courses after beginning nivolumab (Fig. 7). Currently, approximately 3.5 years have passed since beginning nivolumab, and the CEA concentration has normalized to 3.7 ng/mL and CA19-9 to 11.1 U/mL. The most recent CT showed no progression of symptoms; therefore, a complete clinical response was achieved. In addition, the patient developed no side effects during the course of treatment, including immune-responsive adverse events. As a result, her body weight improved significantly to 49 kg, she returned to work, which she could not do previously owing to the side effects of treatment, and her quality of life (QOL) improved. The patient is currently receiving nivolumab, and there is no evidence of recurrence approximately 3 years after starting this therapy (7 years after surgery) (Fig. 8).

## Discussion

Recently, the efficacy of immune checkpoint inhibitors has been demonstrated in various types of cancer. Organisms have an intrinsic immune surveillance mechanism that recognizes and removes cell that are proliferating abnormally. However, precancerous cells are not recognized and removed. If the abnormally proliferating cells acquire the ability to evade immune surveillance, they proliferate and manifest as cancer [6]. The immune checkpoint mechanism that targets cancer cells comprises various types of immune cells that prevent an excessive immune response. One of these mechanisms involves PD-1/programmed death ligand-1 (PD-L1). PD-L1 binds to the PD-1 receptor expressed on T-cells, thereby maintaining a balance between the various immune mechanisms. To avoid triggering the mechanism responsible for overexpression of cancer cells that express PD-L1, the ligand binds the PD-1 receptor on T-cells and suppresses their activation. The interaction between PD-1 and PD-L1 plays an important role in the immune escape mechanism [7], and the anti-PD-1 antibody exerts an antitumor effect by preventing the binding of these two entities [8]. In 2014, the anti-PD-1 antibody, nivolumab, was approved for use in malignant melanoma. Since then, the results of various clinical trials have been reported. Based on the results of the international joint phase III controlled trial, ATTRACTION-2 [9], reported in 2017, nivolumab was also approved for gastric cancer. The effects were reported in the ATTRACTION-2 trial, with a median overall survival of 5.3 months, median progression-free survival of 1.6 months, and a response rate of 11.2%. In the 2-year observation report of the ATTRACTION-2 trial, the median survival time of responding patients with a CR or partial response (PR) was 26.6 months, the 12-month survival rate was 87.1%, and the 24-month survival rate was 61.3%, which were very good [9]. Characteristics and biomarkers in successful and long-term survival cases have not been identified, although the neutrophil-lymphocyte ratio (NLR), PD-L1 expression in tumor cells, and microsatellite instability (MSI) have been reported [10–13]. It has also been reported that MSI and combined positive score (CPS) are good predictors of the therapeutic efficacy of pembrolizumab, another anti-PD 1 antibody [14]. However, in our case, the tumor was not accompanied by microsatellite instability and the PDL1 CPS score was 1 or less. Therefore, these results suggest that other factors are involved as therapeutic effect predictors.

Our patient achieved CR with nivolumab administration; however, the treatment period after achieving CR and the treatment period at the time of recurrence are undetermined, and we are considering how long to continue administering nivolumab. There are reports of recurrence in 3 out of 22 patients who achieved a complete pathological response to chemotherapy [15]. In this case, we considered discontinuing nivolumab administration in the third year, but we continued because the patient was worried about recurrence and wanted to continue nivolumab therapy. We are now considering whether to stop nivolumab treatment in the fourth year. This will be decided in consultation with the patient. Therefore, although CR induced by drug treatment is associated with a better prognosis for patients, we hope that future case accumulation and reporting will provide a definitive policy.

Although no adverse events were observed in our patient, we consider that nivolumab is associated with immune-related adverse events, potentially triggered by T-cell activation, that are not recognized in conventional medicine. The most common symptoms are dermatologic but may include diabetes, diarrhea, thyroid dysfunction, and interstitial pneumonia. However, early diagnosis is difficult because the initial symptoms resemble those commonly associated with cancer, such as malaise and fever. Careful monitoring for these adverse events is necessary during follow-up given that they can occur from several weeks to several years after initiating treatment [16]. At our hospital, we monitor patients treated with nivolumab for immune-related adverse events by performing regular thyroid function tests and other examinations, as appropriate.

## Conclusions

This case is considered a valuable case in which CR was obtained after nivolumab administration. However, it is considered necessary to accumulate and analyze additional cases, such as patients who respond to nivolumab, and determine treatment strategies after the response.

## Data Availability

Not applicable.

## Abbreviations

QOL: Quality of life
HER2: Human epidermal growth factor receptor 2
CT: Computed tomography
PD-1: Anti-programmed cell death 1
UICC: Union for International Cancer Control
PET-CT: Positron emission tomography-computed tomography
PD-L1: Programmed death ligand-1
CR: Complete response
PR: Partial response
NLR: Neutrophil–lymphocyte ratio
MSI: Microsatellite instability
CPS: Combined positive score

## References

[1] Brahmer J, Reckamp KL, Baas P, Crinò L, Eberhardt WE, Poddubskaya E, et al. Nivolumab versus docetaxel in advanced squamous-cell non-small-cell lung cancer. N Engl J Med. 2015;373:123–35. 10.1056/NEJMoa1504627 PMC4681400 26028407

[2] Borghaei H, Paz-Ares L, Horn L, Spigel DR, Steins M, Ready NE, et al. Nivolumab versus docetaxel in advanced non squamous non-small-cell lung cancer. N Engl J Med. 2015;373:1627–39. 10.1056/NEJMoa1507643 PMC5705936 26412456

[3] Brierley JD, Gospodarowick MK, Wittekund C. TNM classification of malignant tumours. 8th ed. New York: Wiley; 2017.

[4] Eisenhauer EA, Therasse P, Bogaerts J, Schwartz LH, Sargent D, Ford R, et al. New response evaluation criteria in solid tumours: revised RECIST guideline (version 1.1). Eur J Cancer. 2009;45:228–47. 10.1016/j.ejca.2008.10.026 19097774

[5] Common terminology criteria for adverse events (CTCAE) v5. 0. National Cancer Institute; 2017.

[6] Dunn GP, Bruce AT, Ikeda H, Old LJ, Schreiber RD. Cancer immunoediting: from immunosurveillance to tumor escape. Nat Immunol. 2002;3:991–8. 10.1038/ni1102-991 12407406

[7] Iwai Y, Ishida M, Tanaka Y, Okazaki T, Honjo T, Minato N. Involvement of PD-L1 on tumor cells in the escape from host immune system and tumor immunotherapy by PD-L1 blockade. Proc Natl Acad Sci USA. 2002;99:12293–7. 10.1073/pnas.192461099 PMC129438 12218188

[8] Topalian SL, Hodi FS, Brahmer JR, Gettinger SN, Smith DC, McDermott DF, et al. Safety, activity, and immune correlates of anti-PD-1 antibody in cancer. N Engl J Med. 2012;366:2443–54. 10.1056/NEJMoa1200690 PMC3544539 22658127

[9] Kang YK, Boku N, Satoh T, Ryu MH, Chao Y, Kato K, et al. Nivolumab in patients with advanced gastric or gastro-oesophageal junction cancer refractory to, or intolerant of, at least two previous chemotherapy regimens (ONO-4538-12, ATTRACTION-2): a randomized, double-blind, placebo-controlled, phase3 trial. Lancet. 2017;390:2461–71. 10.1016/S0140-6736(17)31827-5. 28993052

[10] Shiraishi T, Kano M, Sakata H, et al. Successful treatment of gastric cancer with conversion surgery after nivolumab treatment—a case report. Jpn J Cancer Chemother. 2019;46:1614–6. 31631152

[11] Sacdalan DB, Lucero JA, Sacdalan DL. Prognostic utility of baseline neutrophil-to-lymphocyte ratio in patients receiving immune checkpoint inhibitors: a review and meta-analysis. Onco Targets Ther. 2018;11:955–65. 10.2147/OTT.S153290 PMC5827677 29503570

[12] Namikawa T, Ishida N, Tsuda S, et al. Successful treatment of liver metastases arising from early gastric cancer achieved clinical complete response by nivolumab. Surg Case Rep. 2018;4:71. 10.1186/s40792-018-0479-3 PMC6033840 29978335

[13] Kashima S, Tanabe H, Tanino M, et al. Lymph node metastasis from gastroesophageal cancer successfully treated by nivolumab: a case report of a young patient. Front Oncol. 2019;9:1375. 10.3389/fonc.2019.01375 PMC6927466 31921639

[14] Kim ST, Cristescu R, Bass AJ, et al. Comprehensive molecular characterization of clinical responses to PD-1 inhibition in metastatic gastric cancer. Nat Med. 2018;24:1449–58. 10.1038/s41591-018-0101-z 30013197

[15] Cho H, Nakamura J, Asaumi Y, et al. Long-term survival outcomes of advanced gastric cancer patients who achieved a pathological complete response with neoadjuvant chemotherapy: a systematic review of the literature. Ann Surg Oncol. 2015;22:787–92. 10.1245/s10434-014-4084-9 25223927

[16] Byun DJ, Wolchok JD, Rosenberg LM, Girotra M. Cancer immunotherapy-immune checkpoint blockade and associated endocrinopathies. Nat Rev Endocrinol. 2017;13:195–207. 10.1038/nrendo.2016.205 PMC5629093 28106152

